# Gene expression patterns associated with multidrug therapy in multibacillary leprosy

**DOI:** 10.3389/fcimb.2022.917282

**Published:** 2022-07-22

**Authors:** Helen Ferreira, Thyago Leal-Calvo, Mayara Abud Mendes, Charlotte Avanzi, Philippe Busso, Andrej Benjak, Anna Maria Sales, Cássio Porto Ferreira, Márcia de Berrêdo-Pinho, Stewart Thomas Cole, Euzenir Nunes Sarno, Milton Ozório Moraes, Roberta Olmo Pinheiro

**Affiliations:** ^1^ Leprosy Laboratory, Oswaldo Cruz Institute, Oswaldo Cruz Foundation, Rio de Janeiro, Brazil; ^2^ Global Health Institute, École Polytechnique Fédérale de Lausanne, Lausanne, Switzerland; ^3^ Institut Pasteur, Paris, France; ^4^ Cellular Microbiology Laboratory, Oswaldo Cruz Institute, Oswaldo Cruz Foundation, Rio de Janeiro, Brazil

**Keywords:** multibacillary leprosy, multidrug therapy, lipid metabolism, bacillary load, gene signature

## Abstract

Multidrug therapy (MDT) has been successfully used in the treatment of leprosy. However, although patients are cured after the completion of MDT, leprosy reactions, permanent disability, and occasional relapse/reinfection are frequently observed in patients. The immune system of multibacillary patients (MB) is not able to mount an effective cellular immune response against *M. leprae*. Consequently, clearance of bacilli from the body is a slow process and after 12 doses of MDT not all MB patients reduce bacillary index (BI). In this context, we recruited MB patients at the uptake and after 12-month of MDT. Patients were stratified according to the level of reduction of the BI after 12 doses MDT. A reduction of at least one log in BI was necessary to be considered a responder patient. We evaluated the pattern of host gene expression in skin samples with RNA sequencing before and after MDT and between samples from patients with or without one log reduction in BI. Our results demonstrated that after 12 doses of MDT there was a reduction in genes associated with lipid metabolism, inflammatory response, and cellular immune response among responders (*APOBEC3A, LGALS17A, CXCL13, CXCL9, CALHM6*, and *IFNG*). Also, by comparing MB patients with lower BI reduction versus responder patients, we identified high expression of *CDH19, TMPRSS4, PAX3, FA2H, HLA-V, FABP7*, and *SERPINA11* before MDT. From the most differentially expressed genes, we observed that MDT modulates pathways related to immune response and lipid metabolism in skin cells from MB patients after MDT, with higher expression of genes like *CYP11A1*, that are associated with cholesterol metabolism in the group with the worst response to treatment. Altogether, the data presented contribute to elucidate gene signatures and identify differentially expressed genes associated with MDT outcomes in MB patients.

## Introduction

Leprosy is a chronic infectious disease caused by *Mycobacterium leprae* or *M. lepromatosis*. Multidrug therapy (MDT) (Jakeman and Smith, 1994; Cunha et al., 2004) has been described as effective in reducing the prevalence of leprosy globally. However, there is no convincing evidence for the efficacy of MDT in interrupting the transmission, since a steady number of new patients were reported every year in the last decade (Richardus and Habbema, 2007). A previous study has demonstrated that *M. leprae* drug resistant isolates contribute to leprosy relapse in Brazil and alternative explanations have been proposed such as bacterial persistence, immunosuppression of the host, and reinfection (Kaimal and Thappa, 2009; da Silva Rocha et al., 2012).

Multibacillary (MB) leprosy is characterized by a low cellular immune response against the bacilli and histologically skin lesions of MB patients present an infiltrate that is composed mainly by highly parasitized macrophages, few lymphocytes and numerous plasma cells (Kaplan et al., 1983; Pinheiro et al., 2018; da Silva Prata et al., 2019).

MB treatment consists of a combination of rifampicin, clofazimine, and dapsone, a combination considered efficient to control the bacillary index (BI) after 12 doses of MDT (WHO, 2014). However, not all patients present a reduction in BI after these 12 doses.

Our previous study demonstrated that PBMC from MB MDT-responders (MDT-R, with reduction of at least one log in BI after MDT) produced higher levels of *CXCL10* in response to *M. leprae* when compared to cells from MDT-non-responders (MDT-NR, reduction lower than 1 log in BI after 12-doses MDT) MB patients (Ferreira et al., 2021). Here, we used host transcriptomic profiling by RNA-seq to explore differences in genes and pathways associated with a better response to treatment that could help suggest adjuvant or new and more effective anti-leprosy drugs. Our data points to host pathways that could be associated with a positive outcome to leprosy treatment.

## Materials and methods

### Ethics statement

This study was carried out following institutional research ethics committee approval and in Resolution 466/12 of the National Health Council (CAAE 76328517.2.0000.5248, approval number 2.450.910). All volunteers agreed to participate and signed a free and informed consent before their inclusion in the study covering the longitudinal sample collection. All the patients received clinical treatment, follow-up appointments, and all information, regardless of their participation or exclusion from the study.

### Patient population

A total of 14 adult patients were included, being 10 for RNA-seq and 14 for RT-qPCR analysis. The patients were men between 18 and 68 years old who had been diagnosed with MB leprosy and were categorized according to Ridley and Jopling classification (Ridley and Jopling, 1966) as being lepromatous-lepromatous (LL), with no reaction at the uptake, during or at the completion of treatment (release) (**Table 1**). Other MB patients presenting with borderline leprosy forms (BL and BB) or relapse were excluded to obtain a homogeneous cohort. Patients with comorbidities such as diabetes, hepatitis and diseases caused by other mycobacteria as well as co-infected with the human immunodeficiency virus (HIV) were also excluded. All patients enrolled were treated at the Souza Araújo Outpatient Unit at Fiocruz, Rio de Janeiro, Brazil. Among the recruited patients for RNA-seq, seven received the standard regimen of multidrug therapy (WHO-MDT): rifampicin, dapsone, and clofazimine, and three received an alternative scheme: rifampicin, clofazimine and ofloxacin. Alternatively, to validate the RNA-seq, the data samples from 14 adult patients with LL leprosy were evaluated by RT-qPCR. For RT-qPCR analysis, nine patients received the standard regimen of MDT and five received the alternative scheme. Only patients who took these regimens for twelve months were included. Due the rigor of our inclusion criteria we did not work with a larger cohort, but we selected the most clinically homogeneous cohort in lepromatous group. Skin lesion samples were obtained at diagnosis/before treatment (uptake) and after treatment (release). Skin lesion biopsies were obtained with a six mm punch and cleaved into two longitudinal fragments. One fragment was used for histopathological processing and stained with the Hematoxylin-Eosin and Wade methods for diagnostic purposes, while the second fragment was immediately frozen by immersion in liquid nitrogen and used for transcriptome analysis. After MDT, lesions can be histologically clear according to the BI, which generally decreases around one log per year (Ridley, 1974; Job, 1994; Massone, 2012; Massone et al., 2015). Moreover, for unexplained reasons, some patients do not reduce their BI even after 12 doses of MDT or present only a modest reduction, smaller than one log. To evaluate the profile of immune response associated with a better response to treatment in MB patients, samples were divided into two groups. The first group was composed of patients who presented a reduction in BI at least one log per year (Responders, MDT-R) and the other group was composed of patients that present a reduction lower than 1 log in BI (non-responders, MDT-NR) after the release of 12 doses of MDT (**Table 1**). None of the isolates were resistant and the 1-year follow up demonstrated a reduction in both IB and ILB in both responder and non-responder’s groups.

**Table 1:** Clinical and epidemiological data of cases used in the study.

Case Number	Sex	Age	BI Uptake	BI Release	LBI Uptake	LBI Release	MDTScheme	Group
MB1	M	28	3,5	2,5	1,0	0	Standard	R
MB2	M	43	4,57	4,0	4,7	2,5	Standard	NR
MB3	M	64	5,50	5,0	5,9	5,85	Standard	NR
MB4	M	38	5,0	4,75	4,85	4,8	Standard	NR
MB5	M	47	5,0	3,75	5,85	4,8	Standard	NR
MB6	M	51	4,5	4,0	4,5	4,6	Standard	NR
MB7	M	18	5,5	4,75	5,95	3,6	Standard	NR
MB8	M	68	3,5	2,0	2,6	1,5	Alternative	R
MB9	M	65	3,5	2,25	2,3	3,8	Alternative	R
MB10	M	38	4,0	3,5	3,85	4,6	Alternative	NR
MB11	M	62	2,75	1,25	0	0	Standard	R
MB12	M	28	5,25	4,0	5,85	2,5	Standard	R
MB13	M	41	5,0	4,75	5,7	4,5	Standard	NR
MB14	M	37	5,0	4,25	4,6	2,85	Standard	NR

BI, Bacillary index; LBI, Logaritimic Bacillary index; R, responders; NR, Non-responders.

### RNA isolation

Snap frozen skin biopsies were thawed in wet ice and homogenized in a Polytron Homogenizer PT3100 (Kinematica AG, Switzerland) with TRIzol Reagent. RNA was isolated following the TRIzol Reagent standard manufacturer’s protocol (Ambion, Thermo Fisher Sci., MA, USA). Contaminating DNA was removed using the DNA-free kit according to the manufacturer’s protocol (Thermo Fisher Scientific Inc., MA, USA). RNA integrity was assessed in 1% agarose gel electrophoresis and TapeStation RNA ScreenTape (Agilent Technology, CA, USA).

### Library preparation and illumina mRNA Sequencing

RNA-seq libraries were prepared in two batches and sequenced separately. For the first batch, namely CH, one μg of total RNA was used in library preparation with the Illumina TruSeq mRNA kit (Illumina, USA) and Illumina CD RNA indexes (Illumina, USA), as recommended by the manufacturer. Libraries were quantified and qualified using a qPCR quantification protocol guide (KAPA Library Quantification Kits for Illumina Sequencing platforms) and TapeStation D1000 ScreenTape (Agilent Technologies, USA), respectively. Additional quality control was done using routine agarose electrophoresis and Qubit quantification (Thermo Fisher Sci, MA, USA). The resulting libraries were multiplexed and sequenced using the NextSeq 500 platform (Illumina, USA) for 75 cycles single end. The second library, named USA, was prepared with NEBNext Ultra™ RNA Library Prep Kit (New England Biolabs, MA, USA), and rRNA was removed using NEBNext^®^ Poly(A) mRNA Magnetic Isolation Module (New England Biolabs, MA, USA). Libraries were then sequenced in paired-end mode for 150 cycles with the Illumina NovaSeq 6000 at NovoGene Co.

### RNA-sequencing analysis

RAW bcl files were converted into FASTQ using Illumina’s proprietary script. Read quality was assessed using FastQC v.0.11.8, MultiQC v.1.10, and TrimGalore v.0.6.1. Next, reads were aligned against the human genome hg19 using STAR v.2.6.1d with default settings separately for single-end and paired-end libraries. The number of reads mapping to each gene was estimated using HTSeq v.0.6.1 *(-m union*). Then, count matrices were combined and analyzed in the R 4.1 language and programming environment. The expression of sex-chromosome-specific *UTY* and *XIST* genes was used to rule out sample mislabeling. Multidimensional scaling (MDS) plots were used to inspect the dataset before inferential analyzes. Differential gene expression (DGE) was estimated using DESEq2 v.1.24.0 (*sfType = “poscounts”, minReplicatesForReplace = 3*) after filtering out lowly expressed genes with less than 60 counts across all samples. For comparing the effect of MDT treatment within patients the model included the patient id as a blocking variable. As for the between-individual comparisons, only the CH batch was used including only the independent variable “responder”. The log fold-change estimates were shrunken using the “apeglm” algorithm. Then, nominal P-values were inspected with histograms and adjusted for multiple testing according to Benjamini and Hochberg’s method to control the false discovery rate (FDR). Genes were called differentially expressed (DE) if |log_2_FC| ≥ 1 and FDR ≤ 0.1. For visualization, normalized counts were used in log2 space with ggplot2 v.3.3.0. Hierarchical clustering and heatmaps were done in pheatmap v.1.0.12 using variance stabilized data with gene-wise scaling and centering (z-score) and Euclidean or Ward distances with average or complete agglomeration methods. Gene set enrichment analysis (GSEA) and overrepresentation analysis (ORA) were used for Gene Ontology (GO) and Reactome annotations with clusterProfiler v.3.12.0 and org.Hs.eg.db v.3.8.2. P-values for enrichment analyses were all adjusted for multiple testing using the Benjamini-Hochberg method and FDR at 0.1. Raw data, raw counts, and normalized expression matrices are available in EMBL-EBI Array Express under accession E-MTAB-11605.

### RT-qPCR

RNA was extracted from skin lesion fragments by the TRIzol method (Life Technologies, #15596-018), following the manufacturer’s instructions. To avoid genomic DNA contamination, the RNA was treated with DNAse (RTS DNase Kit, MO BIO Laboratories). RNA integrity was analyzed *via* 1.2% agarose gel electrophoresis. The SuperScript III First-Strand Synthesis System (Life Technologies, 18080-051) was used for reverse transcription. mRNA expression of *CXCL9, CXCL10*, *IFNG, AIM2, OAS1* and *PTX3* were evaluated using TaqMan Fast Universal PCR Master Mix (2X) (Applied Biosystems, #4352042) in a Step One Plus real-time PCR system (Applied Biosystems, MA, USA). All primers and probes were acquired from Thermo Fisher Scientific (#4331182). The 2^−ΔCT^ method was used to analyze gene expression data using glyceraldehyde-3-phosphate dehydrogenase (*GAPDH*; Hs02758991_g1, Thermo-Fisher Scientific) as a reference gene.

### Statistical analysis of RT-qPCR

DGE of RT-qPCR data was estimated using Wilcoxon signed rank test using GraphPad Prism 8. Genes with p-value below 0.05 were considered significantly differentially expressed.

## Results

### Differentially expressed genes after MDT in MB Leprosy

**Figure 1:**
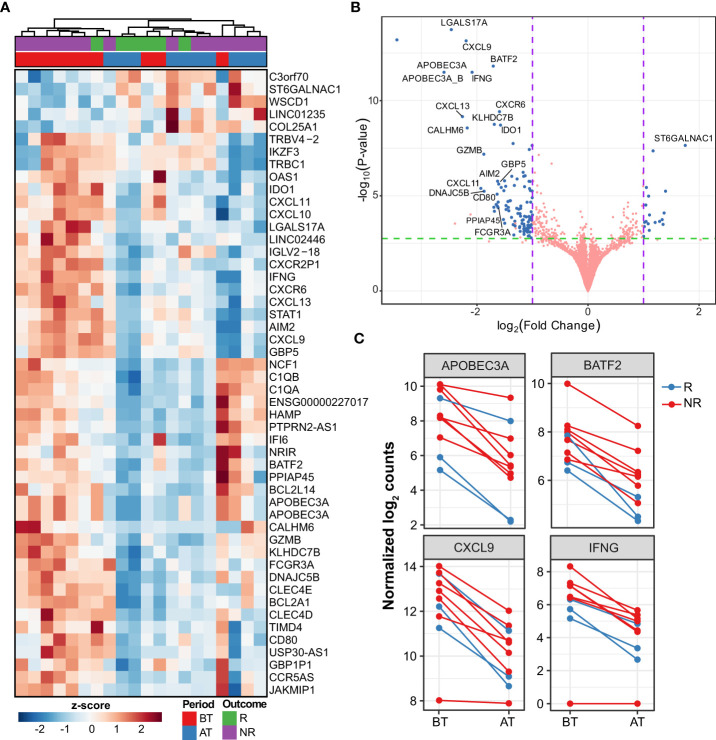
Differentially expressed genes after multi-drug therapy of MB leprosy cases. **(A)** Heatmap depicting top 50 DEG (greatest |log_2_FC| and FDR ≤ 0.1) after comparing after treatment (AT) vs. before treatment (BT). Z-score represents the number of standard deviations away from normalized log_2_ gene expression. Samples (columns) were clustered using hierarchical clustering with Euclidean distance and complete agglomeration. Genes without an official HGNC symbol are shown as their ENSEMBL identifiers. **(B)** Volcano plot showing 121 DEG (blue points) with |log_2_FC| ≥ 1 and false discovery rate (FDR) ≤ 0.1. Genes with the greatest mean differences are shown for emphasis. **(C)** Examples of regulated genes before (BT) after MDT (AT) in MB patients colored according to their responsive (R) or non-responsiveness (NR) to MDT.

The first analysis compared the differential expression between skin lesions of MB patients before and after 12 months of MDT. Patients that developed reactions during this time were excluded from the study to avoid confusion with the effect of thalidomide and other anti-inflammatory drugs. At the end of 12 months, 121 genes (102 down- and 15 upregulated) were differentially expressed (DE) in skin lesions of MB patients (|log_2_FC| ≥ 1 and FDR ≤ 0.1). Of these downregulated genes, six had more than 4-fold reduction compared to before treatment, namely *APOBEC3A*, *LGALS17A*, *CXCL13*, *CXCL9*, *CALHM6*, and *IFNG* (**Figures 1A–C**, **Supplementary Table 1**). Conversely, 15 genes were upregulated at the end of MDT although with less pronounced effects, including *ST6GALNAC1*, *BCL6B*, *COL25A1, DLL4*, and *LINGO1* (**Figures 1A–C**, **Supplementary Table 1**).

**Figure 2:**
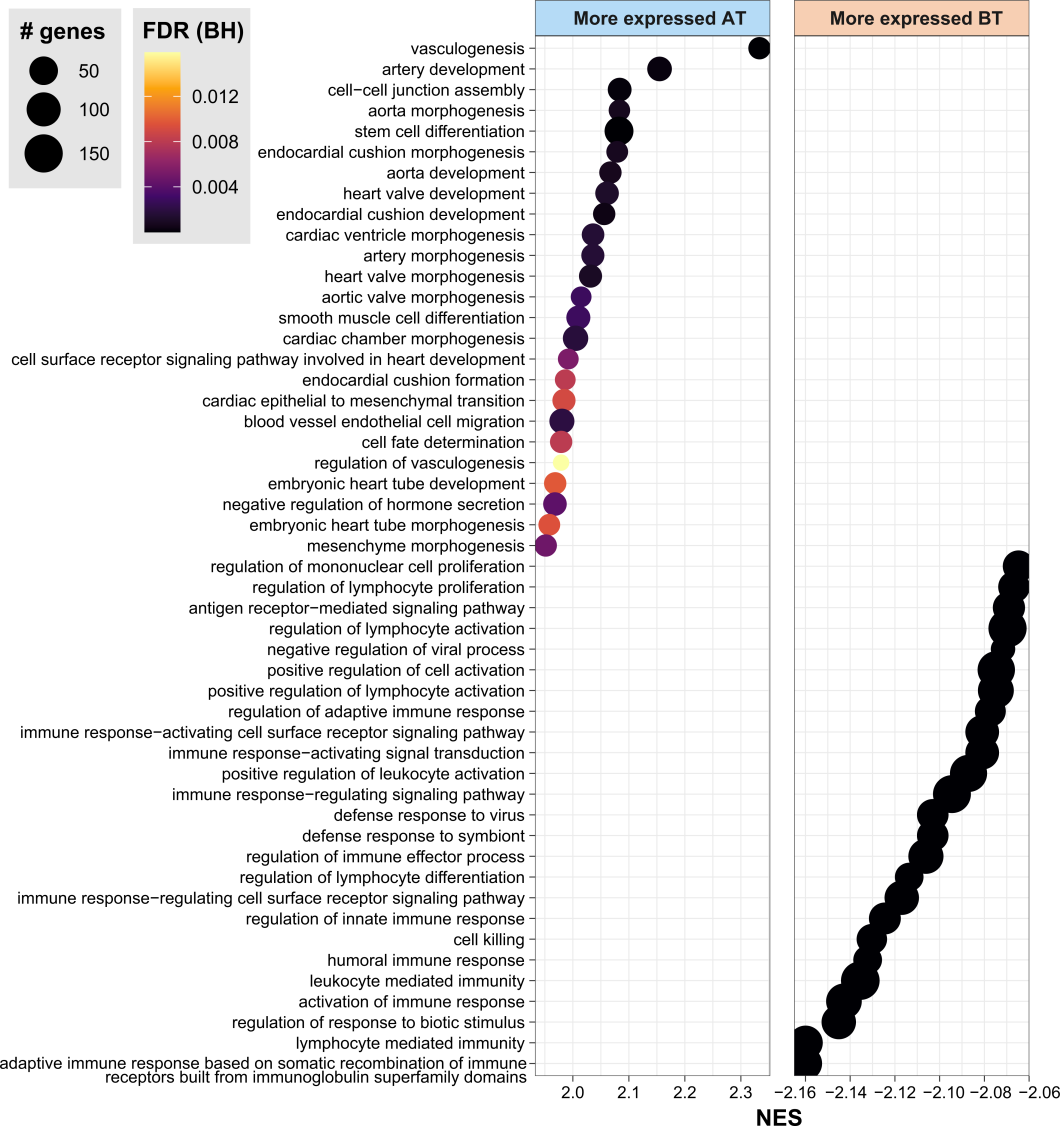
Gene Ontology Biological Processes associated with DEG by GSEA. Gene set enrichment analysis (GSEA) highlighting the top 30 biological processes from Gene Ontology enriched in DEG between AT vs. BT. FDR, false discovery rate. BH, Benjamini-Hochberg. AT, after treatment; BT, before treatment. NES, normalized enrichment score. See also **Supplementary Table 1**.

Next, to contextualize the overall role of these genes, GSEA analysis was done according to the Gene Ontology (GO) and Reactome annotations. As a result, the GO biological processes representative of the genes more expressed before MDT include immune response, neutrophils, lymphocyte differentiation, T cell activation, adaptive immune response, and regulation of innate immune response (**Figure 2**, **Supplementary Table 2**). As for Reactome pathways, neutrophil degranulation, necrosis, phagocytosis, non-canonical NF-κB, and interferon-alpha/beta and gamma were all associated with DEG in after *vs.* before treatment (**Supplementary Figure 1**). Interestingly, compared to before MDT, genes upregulated after MDT were involved with vasculogenesis, endothelial cell differentiation, appendage development, limb development, axonogenesis, and epidermis development, which are consistent with recovering skin homeostasis (**Figure 2**, **Supplementary Table 2**).

### Differentially expressed genes among MDT-R and MDT-NR

**Figure 3:**
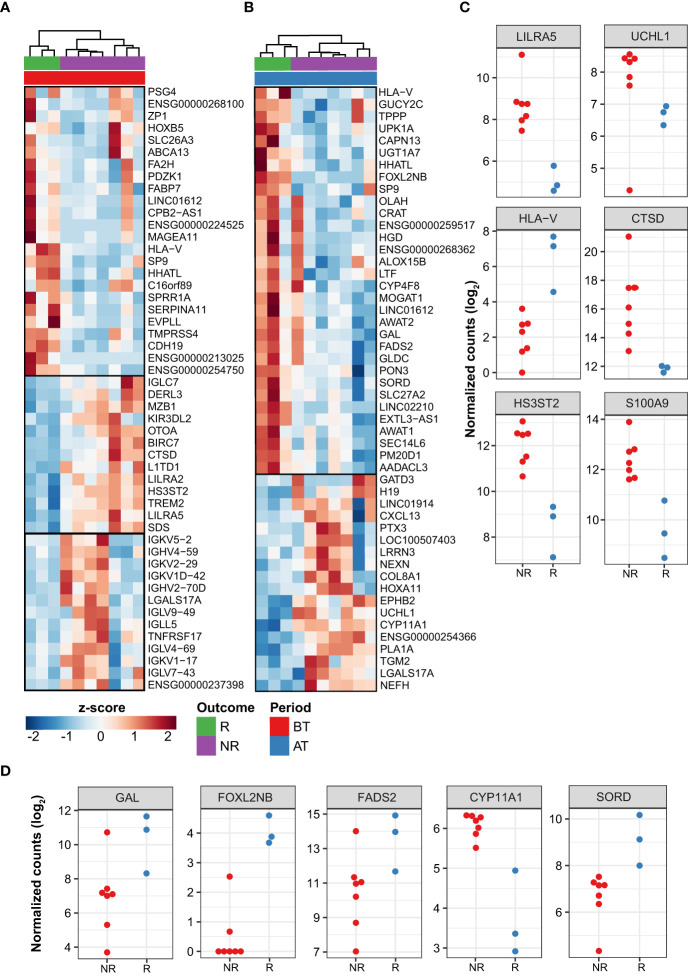
Differentially expressed genes between patient responses to MDT. Top 50 DEG from responder (R) vs. non-responder (NR) LL patients before treatment **(A)** and after treatment **(B)**. Z-score represents the number of standard deviations away from normalized log_2_ gene expression. Top 50 genes with greatest |log_2_FC| and FDR ≤ 0.1 are shown. Samples (columns) were clustered using hierarchical clustering with Euclidean distance and complete agglomeration. Genes without an official HGNC symbol are shown as their ENSEMBL identifiers. Plot showing the expression of a subset of DEG with distinct patterns between R and NR patients either before **(C)** or after treatment **(D)**. In C-D, only the subset of samples from sequencing batch “CH” is shown as used in DESeq2’s generalized linear model (see Methods).

After 12-doses of MDT MB patients are considered cured, although a high proportion of patients do not significantly reduce their BI after this period. Then, we compared MDT-R *vs.* MDT-NR before and after MDT. Before MDT, there were 299 (181 up- and 118 downregulated) DEG between MDT-R and MDT-NR (|log_2_FC| ≥ 1 and FDR ≤ 0.1). On average, compared to MDT-NR, the expression of *CDH19*, *TMPRSS4, PAX3, FA2H, HLA-V*, *FABP7*, and *SERPINA11* was higher in MDT-R (**Supplementary Table 3**, **Figures 3A–C**). Enrichment analysis using ORA revealed that upregulated genes in MDT-R *vs.* MDT-NR before treatment were involved with skin homeostasis and development, fat-soluble vitamin metabolism, mesenchymal and stem cell maintenance, and semaphorin-plexin signaling pathway (**Supplementary Figure 2**, **Supplementary Table 4**). Conversely, before MDT, MDT-NR had a higher expression of *S100A9, LILRA5, DERL2, CTSD, FCGR1A, HS3ST2*, *TREM2*, and many immunoglobulin-related genes than MDT-R (**Figures 3A–C**, **Supplementary Table 3**).

The number of modulated genes between MDT-R vs. MDT-NR was smaller after MDT where a total of 53 genes were DE (35 up- and 18 downregulated). Among the genes upregulated in MDT-R after MDT were *LTF, HLA-V, GAL, FADS2, FOXL2BNB, SORD, MOGAT1* among others (**Figures 3B–D**, **Supplementary Table 5**). Over-representation analysis of these upregulated genes showed marked involvement with lipid metabolism (**Supplementary Figure 3**, **Supplementary Table 6**). However, after MDT, MDT-NR had higher expression of *PTX3, CXCL13, UCHL1, TGM2*, and *CYP11A1* than MDT-R (**Figures 3B–D**, **Supplementary Table 5**).

Genes identified as differentially expressed by RNA-seq were selected for replication by real time RT-qPCR based on empirical criteria such as the observation of a larger log fold change and/or the inclusion of the gene in a pathway or enriched biological process. In this experiment, four independent samples were tested together with 10 previous samples used in RNA-seq analysis. Six genes were selected: *CXCL9*, *CXCL10*, *IFNG*, *AIM2*, *OAS1* and *PTX3*. The expression of these genes was validated by real time RT-qPCR, with a result quite similar to the RNAseq, although not all genes were statistically significant. A significant difference was observed in *IFNG, CXCL10* and *AIM2* in the non-responder’s group when comparing before and after treatment (**Supplementary Figure 4**).

## Discussion

MDT has been effective against leprosy and patients are cured after the completion of MDT with low rates of relapses (Nery et al., 2021). Nevertheless, leprosy reactions and permanent disability have been observed in many patients (White and Franco-Paredes, 2015; Nery et al., 2021). In this scenario, it is important to better understand why some patients do not have a significant decrease in BI levels, especially because this can be a risk factor for reactional states and consequently high morbidity after release.

The complex interplay between innate and adaptive immune response during MB leprosy influences the progression of leprosy disease (van Hooij and Geluk, 2021) and likely the ability to clear the bacilli during treatment. Macrophages from MB patients present an alternative phenotype, which is associated with a more susceptible profile to *M. leprae* infection (Montoya et al., 2009; Moura et al., 2012; de Oliveira Fulco et al., 2014; de Mattos Barbosa et al., 2017). Here, we identified a molecular signature associated with a better response to MDT. Analysis of gene expression after MDT revealed a significant decrease in the expression of 102 genes after 12 doses of MDT. To avoid any influence of inflammatory response and/or the use of corticosteroids in the pattern of gene expression, we selected samples from lepromatous lepromatous patients that have taken 12 doses in 12 months without reaction.

Between the genes that were down regulated after MDT we can observe chemokines like *CXCL9* and *CXCL13*. The CXCL-13 is a chemokine involved in chemotaxis of B cells and known as B-lymphocyte chemoattractant (BLC). Tió-Coma and colleagues (Tió-Coma et al., 2019) described that the gene *CXCL13* can distinguish leprosy contacts from leprosy patients, corroborating our data of increased *CXCL13* expression during the active disease.

A study in Vietnamese patients showed upregulation of IFN pathway genes including IFN-γ and STAT1 in samples from leprosy patients after stimulation of Peripheral Blood Mononuclear Cells (PBMCs) by sonicated antigens (Manry et al., 2017). Here, we observed that MDT was able to significantly decrease the expression of genes of the IFN pathway in cells from MB patients, including *IFNG, STAT1, GBP5, IFI6* and *OAS1*. Guanylate binding protein 5 (GBP5) has been reported to be a critical cellular factor in inflammasome assembly and it induces cytokines like IL-1β and IL-18, through the activation of NLRP3 and AIM2 (Cheng et al., 1983; Schwemmle and Staeheli, 1994). AIM2 decreased significantly in samples from patients after MDT, which can indicate that treatment decreases the expression of IFN-regulated genes and activation of inflammasome AIM2.

Up regulated genes in cells from MDT-R group before MDT are associated with cellular adhesion (*CDH19*) and proliferation, apoptosis, bactericidal (*SERPINA11*), indicating activation of cellular responses associated with improved microbicidal capacity. In MDT-NR group, genes upregulated before treatment were associated with neutrophil chemotaxis (*S100A9*), innate immune responses (*LILRA5*), degradation of misfolded proteins (*DERL2*), protease and hydrolase activity (*CTSD*), transferase activity (*HS3ST2*) and recognition of Fc-gamma (*FCGR1A*). In addition, *TREM2* was up regulated in samples from non-responders’ patients. *TREM2* encodes the protein that acts as a receptor for lipoprotein particles such as LDL, VLDL, and HDL and apolipoproteins. It suppresses PI3K, NF-kB and ERK signaling and increases the expression of IL-10 and TGF-β. IL-10 is a cytokine associated with the induction of a phagocytic and permissive macrophage phenotype in leprosy patients (Montoya et al., 2009; de Oliveira Fulco et al., 2014) and we can suggest that the regulation of *TREM2* after MDT in MB patients may impact the outcome of the treatment.

After MDT, *LTF* is one of the relevant genes upregulated in MDT-R. It encodes the lactotransferrin that is a member of the transferrin family of genes and its protein product is found in the secondary granules of neutrophils. It is associated with iron homeostasis. The role of iron binding proteins in leprosy has been previously described (Moura et al., 2012; de Mattos Barbosa et al., 2017; Pinheiro et al., 2018; da Silva Prata et al., 2019). Other upregulated genes in the MDT-R were involved in nociception (*GAL*), biosynthesis of highly unsaturated fatty acids (*FADS2*), glucose metabolism and lipid biosynthesis (*SORD, MOGAT*).

In MDT-NR group the genes upregulated after treatment were involved in inflammation and complement activation (*PTX3*), chemotaxis of B cells (*CXCL13*), protease activity (*UCHL1, TGM2*) and lipid metabolism (*CYP11A1*). CYP11A1 is a mitochondrial heme protein oxygenase that is involved in the metabolism of cholesterol to pregnenolone (Guryev et al., 2003). *M. leprae* does not use cholesterol as a nutritional source, although cholesterol colocalizes to *M. leprae*-containing phagosomes and the blockade of cholesterol decreases the bacterial survival (Mattos et al., 2014). In lepromatous lesions, host-derived oxidized phospholipids were detected in macrophages, and one specific oxidized phospholipid, 1-palmitoyl-2-(5, 6-epoxyisoprostane E2)-sn-glycero-3-phosphorylcholine accumulate in macrophages infected with live mycobacteria (Cruz et al., 2008).

de Macedo and colleagues demonstrated that differences in lipid signal intensities and localization were observed before and after MDT (de Macedo et al., 2015). After MDT, lipid distribution was like that observed in control skin samples, suggesting the capacity of MDT to modulate the expression of lipids that could be associated with the pathogenesis of the disease. Transcriptomic studies also showed a higher expression of genes involved in lipid metabolism in biopsies obtained from lepromatous leprosy patients (Guerreiro et al., 2013; Leal Calvo and Moraes, 2020). It is known that the breakdown of phospholipids generates free fatty acids and other lipid products involved in signaling pathways. Fatty acids are metabolized into lipid mediators involved in inflammatory processes or act directly on cellular receptors. The data presented here suggest that different metabolic pathways should be modulated in MB patients, but lipid metabolism is a common pathway in both responders and non-responder’s MB patients. Our future studies will evaluate the molecules associated with the lipid metabolism that could be associated with the reduction in bacillary load in the responder’s group.

Rifampicin is the only drug of the components of MDT that displays powerful bactericidal activity against *M. leprae* (Waters et al., 1978; Cambau and Williams, 2019). In the present study, two responder’s patients used ofloxacin that is another powerful bactericidal drug (Cambau and Williams, 2019). The use of a different bactericidal drug in MDT could be considered a bias. However, a previous study demonstrated that the decline of bactericidal index among MB patients in regimens that used rifampicin or ofloxacin had an average of 0.8 plus per year (Khang et al., 2019), demonstrating that the mean decrease of bacterial index per year is the same independently of the bactericidal drug used. Due to the rigor in the inclusion criteria of this study, we acknowledge this study’s small sample and the preliminary nature of our data, but nonetheless set the stage for new studies and questions to be tested in larger cohort groups.

The present study brings new information associated with the host genes and/or pathways modulated by MDT that are associated with a decrease in BI after treatment, which could be used as a target in strategies to identify new molecules to improve the lesion remission and bacilli clearance after discharge that could prevent inflammatory reactions and provide a better quality of life for these patients.

## Data Availability Statement

The RNA-seq data have been deposited in the ArrayExpress database at EMBL-EBI (http://www.ebi.ac.uk/arrayexpress) under accession number E-MTAB-11605.

## Ethics Statement

The studies involving human participants were reviewed and approved by This study was carried out following institutional research ethics committee approval and in Resolution 466/12 of the National Health Council (CAAE 76328517.2.0000.5248, approval number 2.450.910). The patients/participants provided their written informed consent to participate in this study.

## Author contributions

Conceptualization, HF, ES, MOM, and RP; methodology, HF, TL-C, MAM, PB, AB, MBP, SC, MOM., RP. CA; formal analysis, TL-C; investigation, AS, CF, HF, RP; writing, HF, TL-C, MOM, RP; project administration, ES, MOM, RP; funding acquisition, SC, MOM, RP. All authors have read and agreed to the published version of the manuscript.

## Funding

This research was funded by Foundation Carlos Chagas Filho Research Support of the State of Rio de Janeiro (FAPERJ), grant number – E-26/201.176/2021, Brazilian National Council for Scientific and Technological Development (CNPq), grant numbers 303834/2017-0, and 3128021/2020-0. TLC received a Ph.D. scholarship from CNPq from 2018 to 2022, the Fondation Raoul Follereau (SC), the Swiss National Science Foundation grants IZRJZ3_164174 (SC), and the Heiser Program of the New York Community Trust for Research in Leprosy, grant no. P18-000250 (MOM and CA).

## Conflict of interest

The authors declare that the research was conducted in the absence of any commercial or financial relationships that could be construed as a potential conflict of interest.

## Publisher’s note

All claims expressed in this article are solely those of the authors and do not necessarily represent those of their affiliated organizations, or those of the publisher, the editors and the reviewers. Any product that may be evaluated in this article, or claim that may be made by its manufacturer, is not guaranteed or endorsed by the publisher.
