# Supplementary material for: Gene expression patterns associated with multidrug therapy in multibacillary leprosy
Source: Front Cell Infect Microbiol. 2022 Jul 22;12:917282. doi: 10.3389/fcimb.2022.917282 (PMC9354612; doi:10.3389/fcimb.2022.917282)
Supplement: Supplementary file 1 [file DataSheet_1.pdf]

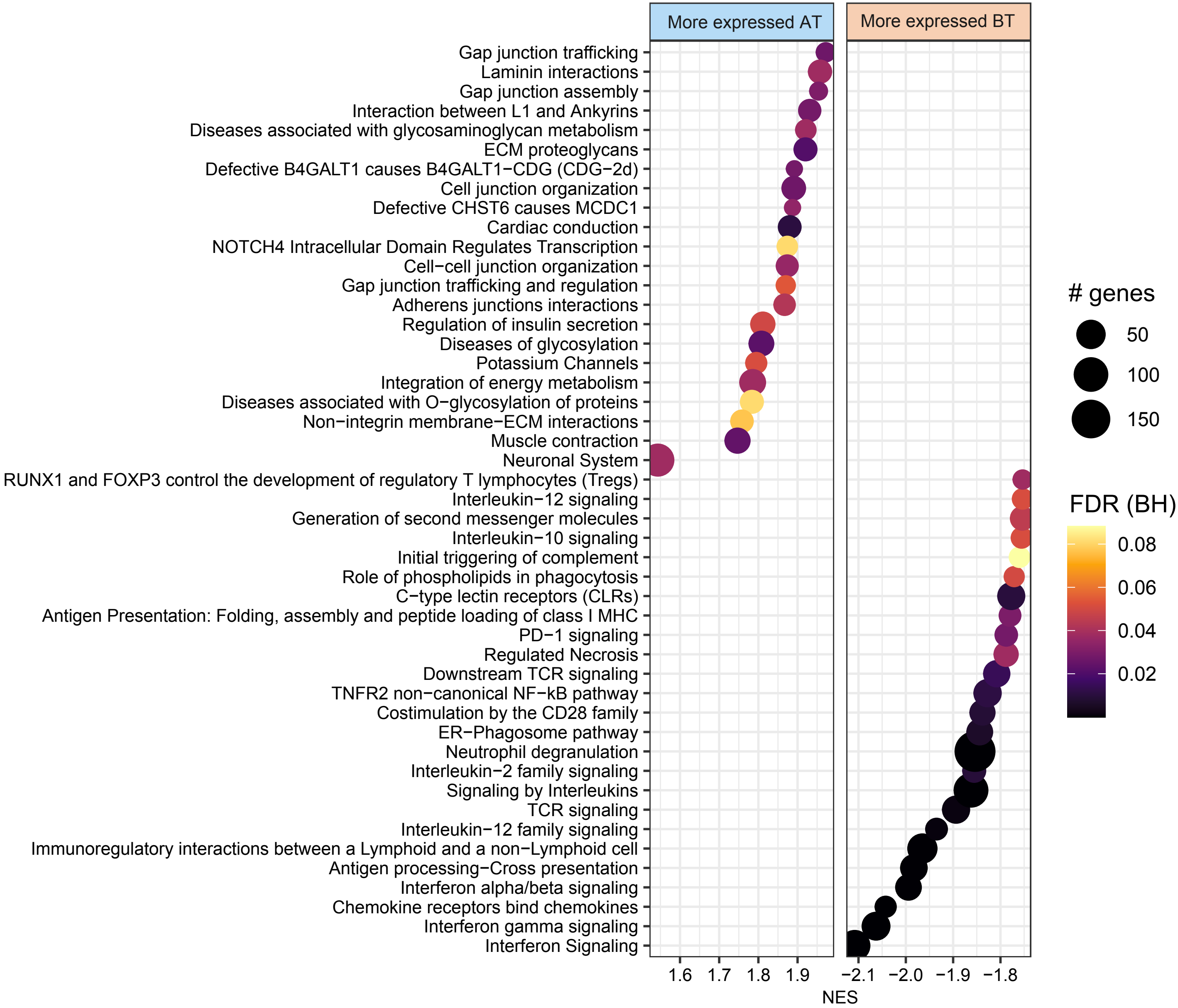

Suppl. Fig. 1. GSEA results according to the Gene Ontology (GO) and Reactome annotations after vs. Before treatment.
