# Supplementary material for: Gene expression patterns associated with multidrug therapy in multibacillary leprosy
Source: Front Cell Infect Microbiol. 2022 Jul 22;12:917282. doi: 10.3389/fcimb.2022.917282 (PMC9354612; doi:10.3389/fcimb.2022.917282)
Supplement: Supplementary file 2 [file DataSheet_2.pdf]

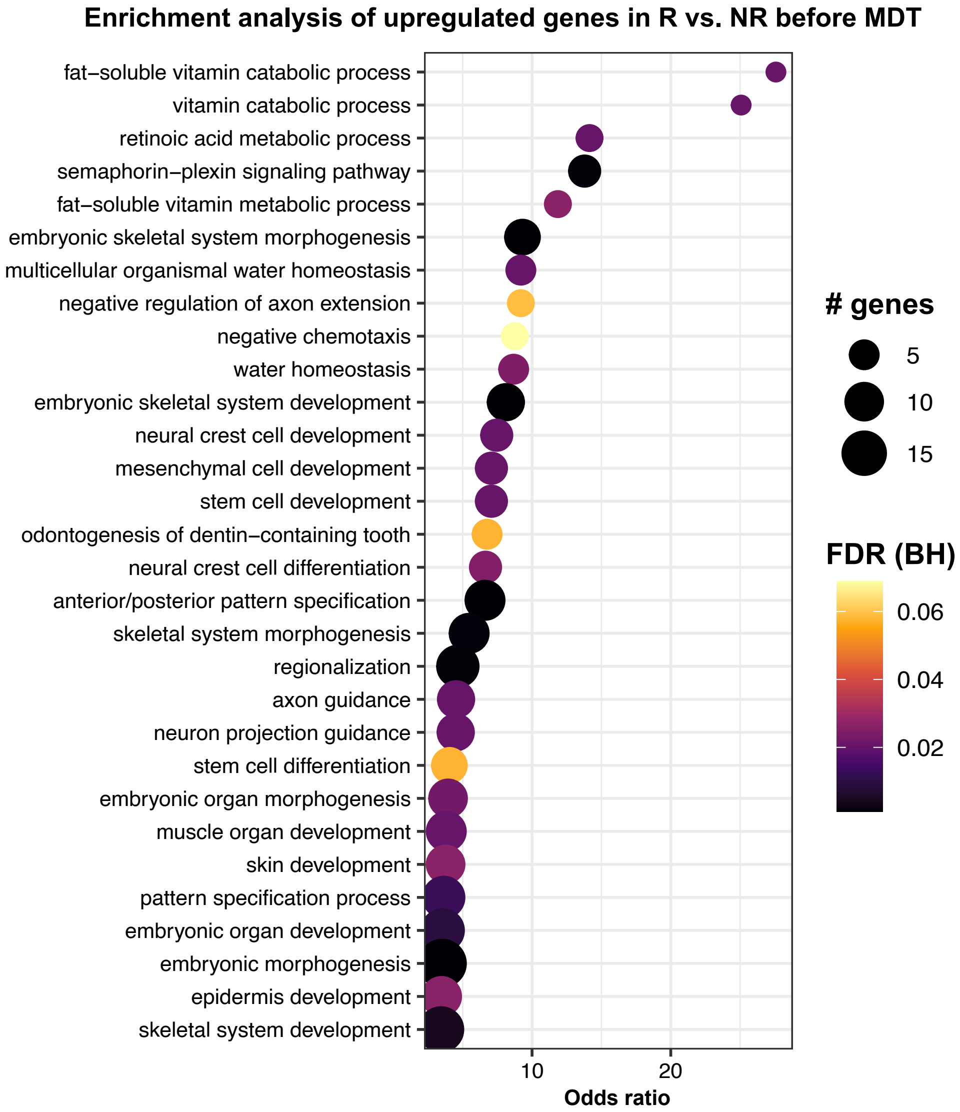

Suppl. Fig. 2. Enrichment analysis in MDT-R vs. MDT-NR before treatment
