# Supplementary material for: Gene expression patterns associated with multidrug therapy in multibacillary leprosy
Source: Front Cell Infect Microbiol. 2022 Jul 22;12:917282. doi: 10.3389/fcimb.2022.917282 (PMC9354612; doi:10.3389/fcimb.2022.917282)
Supplement: Supplementary file 3 [file DataSheet_3.pdf]

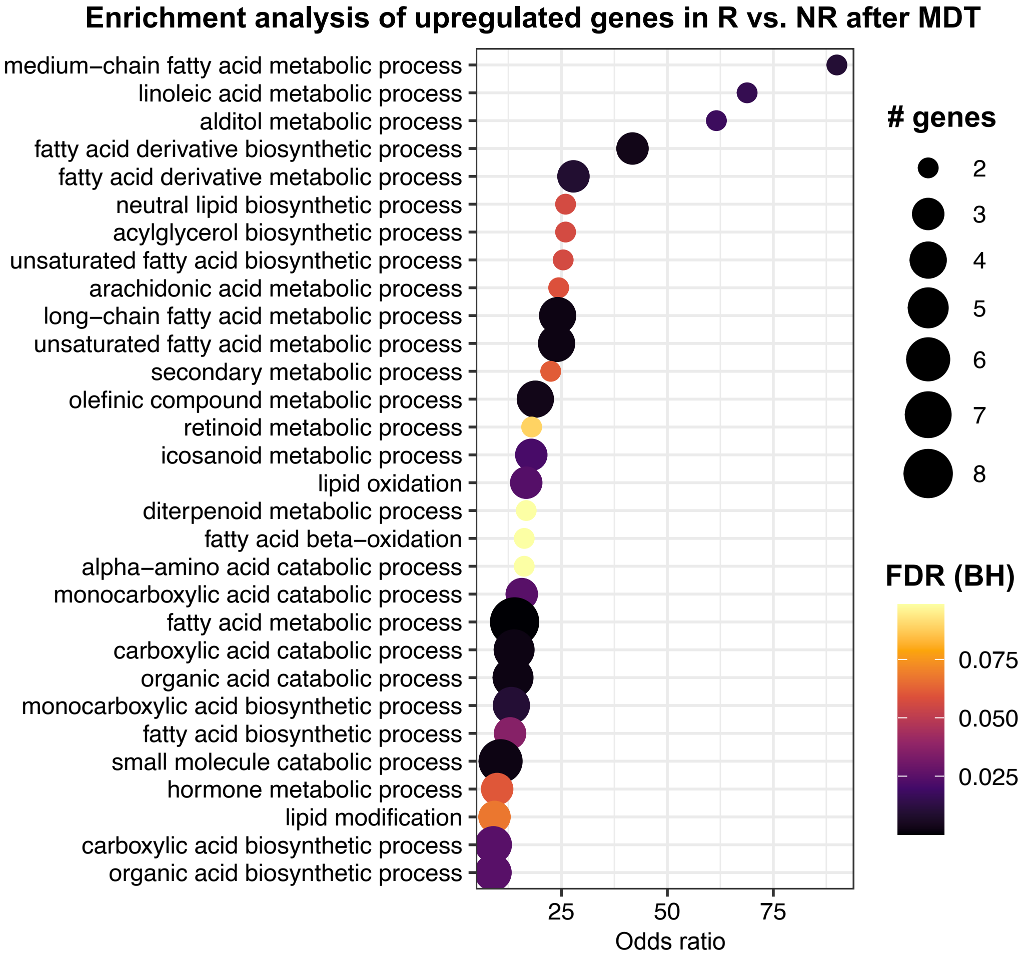

Suppl. Fig. 3. Enrichment analysis in MDT-R vs. MDT-NR after treatment
