# Supplementary material for: Gene expression patterns associated with multidrug therapy in multibacillary leprosy
Source: Front Cell Infect Microbiol. 2022 Jul 22;12:917282. doi: 10.3389/fcimb.2022.917282 (PMC9354612; doi:10.3389/fcimb.2022.917282)
Supplement: Supplementary file 4 [file DataSheet_4.pdf]

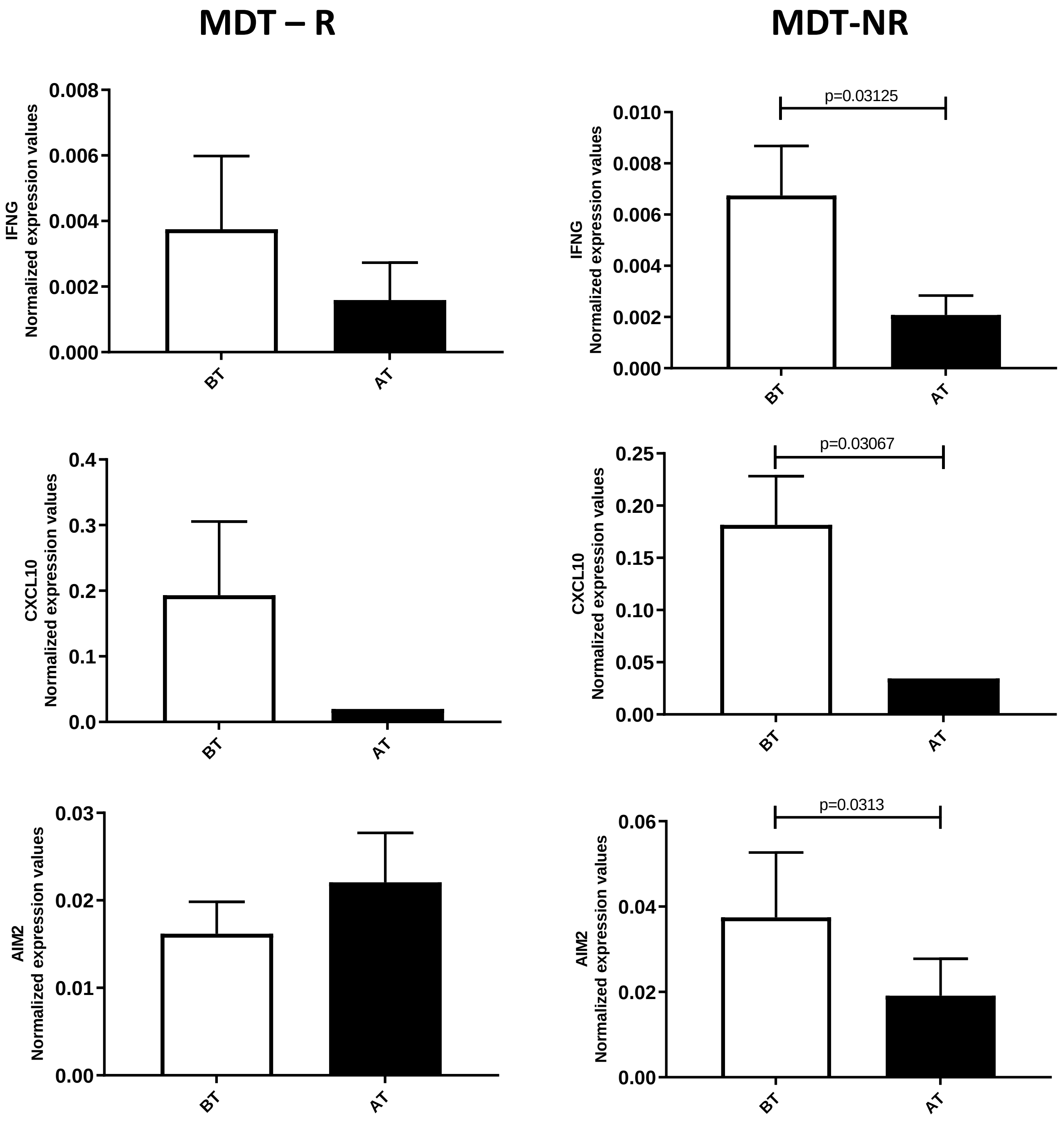

Suppl. Figure 4. RT-PCR analysis of gene expressed before (BT) or After treatment (AT) in the group of responders (MDT-R) and non-responders' MB patients (MDT-NR). MDT-R (n=5), MDT-NR (n=9).
